# Simple fabrication of N-doped mesoporous TiO_2_ nanorods with the enhanced visible light photocatalytic activity

**DOI:** 10.1186/1556-276X-9-34

**Published:** 2014-01-16

**Authors:** Xiufeng Zhou, Juan Lu, Jingjing Jiang, Xiaobin Li, Mengna Lu, Guotao Yuan, Zuoshan Wang, Min Zheng, Hyo Jin Seo

**Affiliations:** 1 College of Chemistry, Chemical Engineering and Materials Science, Soochow University, Suzhou 215123, China; 2 National Engineer Laboratory for Modern Silk, Soochow University, Suzhou 215123, China; 3 Department of Physics and Center for Marine-Integrated Biomedical Technology, Pukyong National University, Busan 608-737, Republic of Korea

**Keywords:** Nanorods, Mesoporous, TiO_2_, Photocatalyst, Visible light

## Abstract

N-doped mesoporous TiO_2_ nanorods were fabricated by a modified and facile sol–gel approach without any templates. Ammonium nitrate was used as a raw source of N dopants, which could produce a lot of gasses such as N_2_, NO_2_, and H_2_O in the process of heating samples. These gasses were proved to be vitally important to form the special mesoporous structure. The samples were characterized by the powder X-ray diffraction, X-ray photoelectron spectrometer, nitrogen adsorption isotherms, scanning electron microscopy, transmission electron microscopy, and UV-visible absorption spectra. The average length and the cross section diameter of the as-prepared samples were *ca*. 1.5 μm and ca. 80 nm, respectively. The photocatalytic activity was evaluated by photodegradation of methylene blue (MB) in aqueous solution. The N-doped mesoporous TiO_2_ nanorods showed an excellent photocatalytic activity, which may be attributed to the enlarged surface area (106.4 m^2^ g^-1^) and the narrowed band gap (2.05 eV). Besides, the rod-like photocatalyst was found to be easy to recycle.

## Background

Since the exciting discovery of the synthesis of TiO_2 - *x*_N_*x*_ film with an enhanced visible light absorption [1], N-doped TiO_2_ nanoparticles have been widely studied in the fields of degrading recalcitrant organic contaminants under visible light in recent years [2,3]. However, practical applications of N-doped TiO_2_ nanoparticles are greatly limited due to their low recycle rate. To solve this problem, N-doped TiO_2_ with different morphologies such as nanowires [4], nanotubes [5], hollow spheres [6], and nanorods were prepared [7,8]. It is well known that N-doped TiO_2_ nanorods can be fabricated by chemically nitriding TiO_2_ nanorods. However, with this route, the nitridation is limited in the surface of the nanorods at a very low level, and thin nitridation layer can be easily removed during the photocatalytic reaction [9]. Besides, the rod-like structure leads to the formation of small surface areas in many cases due to the accumulation of the nanoparticles.

In this work, N-doped TiO_2_ nanorods with mesoporous structure were fabricated by a modified and facile sol–gel approach without any templates. The photocatalytic activity was evaluated by photodegradation of methylene blue (MB) in aqueous solution. The reasons why the N-doped mesoporous TiO_2_ nanorods showed an excellent photocatalytic activity and photochemical stability had been investigated.

## Methods

### Materials

In the experiments, deionized water was used. All of the chemicals were analytical grade. TiO_2_ used for comparison was Degussa P25 (Frankfurt, Germany), whose surface area and particle size were reported as 50 m^2^ g^-1^ and 21 nm, respectively [10].

### Preparation of N-doped mesoporous TiO_2_ nanorods

Typically, 5 mL of tetrabutyl titanate (TBOT), 30 mL of ethanol, and certain ammonium nitrate were mixed together in the reaction flask of the rotary evaporator, and ten agate granules with a diameter of about 1 cm were added into the system for better stirring. The rotary evaporator was turned on and the system was maintained at 25°C. In the mean time, an air blower connected with a round bottom flask containing some deionized water was turned on to transport air at a rate of 40 L min^-1^. A small amount of water vapor was carried into the reaction flask with air to react with the TBOT. The TBOT solution was hydrolyzed slowly to form a cream color emulsion. Reaction stopped after 3 h and then the emulsion was distillated at 50°C for 15 min under vacuum. Finally, the samples were annealed at different temperatures for 2 h to obtain the N-doped mesoporous TiO_2_ nanorods, designated as NMTNR-*x*-*y*, where *x* represents the theoretical molar ratio of N (%) and *y* represents the calcination temperature (°C).

### Characterization of the samples

The crystalline phase identification and structural analysis were carried out by X-ray diffraction (XRD) instrument with Cu Kα radiation. A Japan ULVAC-PHI PHI 5000 VersaProbe X-ray photoelectron spectrometer (XPS; Kanagawa, Japan) was applied to analyze the elemental composition and state of the samples. The microstructures were analyzed by scanning electron microscopy (SEM), transmission electron microscopy (TEM), and high-resolution transmission electron microscopy (HRTEM). N_2_ adsorption-desorption isotherms were measured at 77 K on a Micromeritics Tristar 3020 system (Norcross, GA, USA). The UV-visible (UV–vis) absorbance spectra of the samples were characterized using a Japan Shimadzu UV240 UV–vis spectrophotometer (Kyoto, Japan).

### Photocatalytic activity

The photocatalytic activity of the samples was estimated by MB degradation performed in a 500-mL cylindrical glass photocatalytic reactor, and a 500-W xenon lamp was selected as the visible light source. Between the xenon lamp and reactor, a cut filter was inserted to eliminate ultraviolet light. In a typical experiment, 0.08 g of photocatalyst was dispersed into 250 mL of MB solution (10 mg L^-1^). The actual effect of photocatalytic activity by chemical reaction was studied by maintaining the solutions in the dark for 1 h before irradiation. The MB solution (5 mL) was taken out every 5 min and analyzed using UV–vis spectrophotometer. The degradation of MB can be calculated via the formula *η =* (1 – *A*_*i*_/*A*_0_) × 100%, where *A*_0_ is the absorbance of the original MB solution before irradiation and *A*_i_ is the absorbance of MB solution measured every 5 min. The photodegradation of MB follows pseudo-first-order kinetics. Its kinetics can be expressed as ln(*C*_0_/*C*) *= kt*, where *k* (per minute) is the degradation rate constant.

The stability of photocatalyst was evaluated by the degradation of MB with reused photocatalyst, and 250 mL of new MB solution (10 mg L^-1^) was added into the reactor each time.

## Discussion

**Figure 1:**
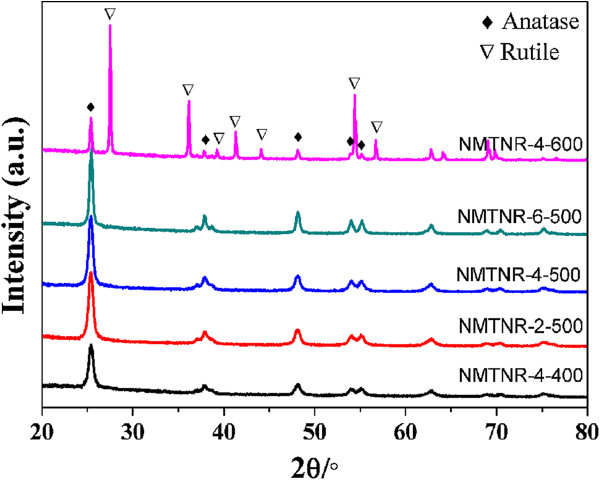
**XRD patterns of N-doped mesoporous TiO**_**2 **_**nanorods.**

Figure 1 shows the typical XRD patterns of N-doped mesoporous TiO_2_ nanorods. It is obvious that the samples except NMTNR-4-600 were in anatase phase according to the identified diffraction peaks (JCPDS no. 21–1272). The weaker peak of NMTNR-4-400 indicates the lower crystallinity of the sample. The average crystal sizes of the samples were calculated with the Scherrer formula and were listed in Table 1. In addition, no nitrogen-derived peaks can be detected in the samples. This is because of the low dosage of the dopant well dispersed in mesoporous TiO_2_ nanorods [11,12].

**Table 1:** Structural properties of the different samples

**Sample**	**Crystal size A/R**^**a **^**(nm)**	**Accurate N content**^**b **^**(at.%)**	***S***_**BET**_^**c **^**(m**^**2**^ **g**^**-1**^**)**	***D***_**p**_^**d **^**(nm)**	***V***_**p**_^**e **^**(cm**^**3**^ **g**^**-1**^**)**	***E***_**g**_^**f **^**(eV)**
NMTNR-4-400	12.7/-	0.74	87.6	6.2	0.1641	2.14
NMTNR-2-500	13.5/-	0.53	83.5	6.5	0.1621	2.23
NMTNR-4-500	15.1/-	0.86	90.1	6.1	0.1623	2.16
NMTNR-6-500	20.6/-	1.31	106.4	9.0	0.2550	2.05
NMTNR-4-600	35.5/58.6	0.32	76.1	7.0	0.1527	2.83

^a^Crystal size of the anatase (A)/rutile (R) particles calculated from XRD results. ^b^Accurate N content (at.%) estimated from XPS. ^c^BET specific surface area. ^d^BJH adsorption average pore diameter (4 V/A). ^e^Single point adsorption total pore volume of pores less than 176.5958 nm diameter at *P*/*P*_0_ = 0.988927610. ^f^The band gap values estimated with Kubelka-Munk function from UV–vis absorbance spectra.

**Figure 2:**
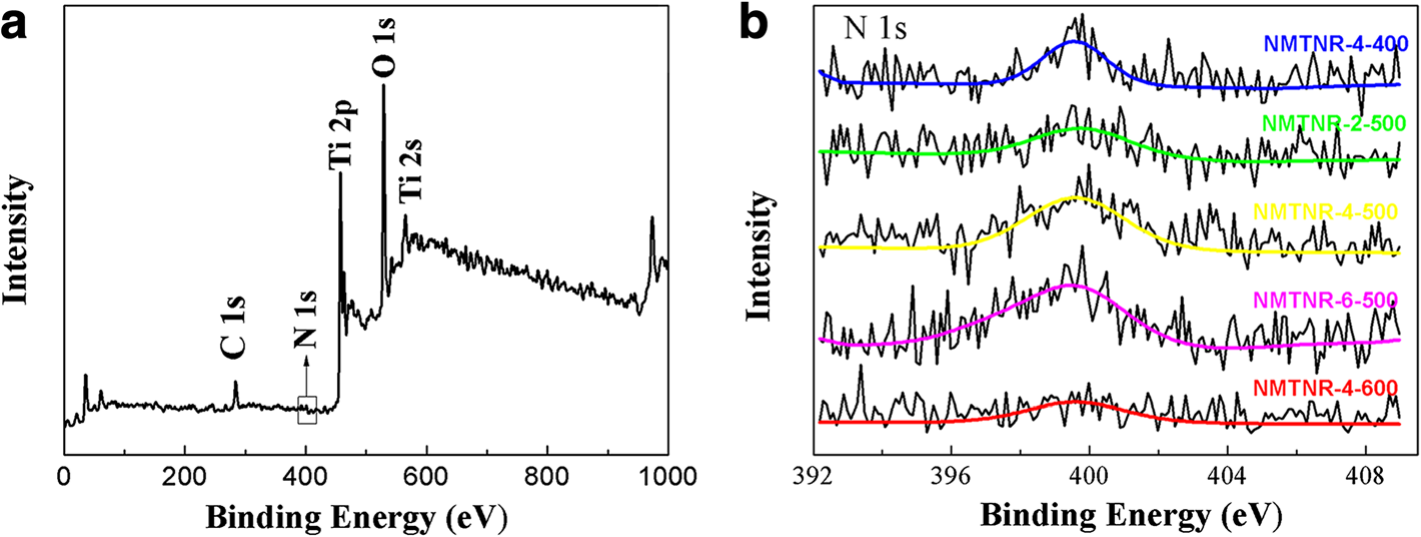
**XPS spectra of NMTNR-4-500 (a) and N 1** ***s *XPS spectra of N-doped mesoporous TiO**_**2 **_**nanorods (b).**

XPS analysis of the sample NMTNR-4-500 was shown in Figure 2a. The binding energies were corrected for specimen charging by referencing C l*s* to 285 eV. The peaks observed in this spectrum were assigned to C, O, Ti, and N. Figure 2b displays the high-resolution N 1 *s* spectra, which reveals a major N 1 *s* peak at around 400 eV due to the adsorbed NO or N in Ti-O-N and O-Ti-N bonds [2,13,14]. The N contents of different samples estimated from XPS spectra were listed in Table 1. It is obvious that the N peaks become stronger and stronger with the increase of the N content.

**Figure 3:**
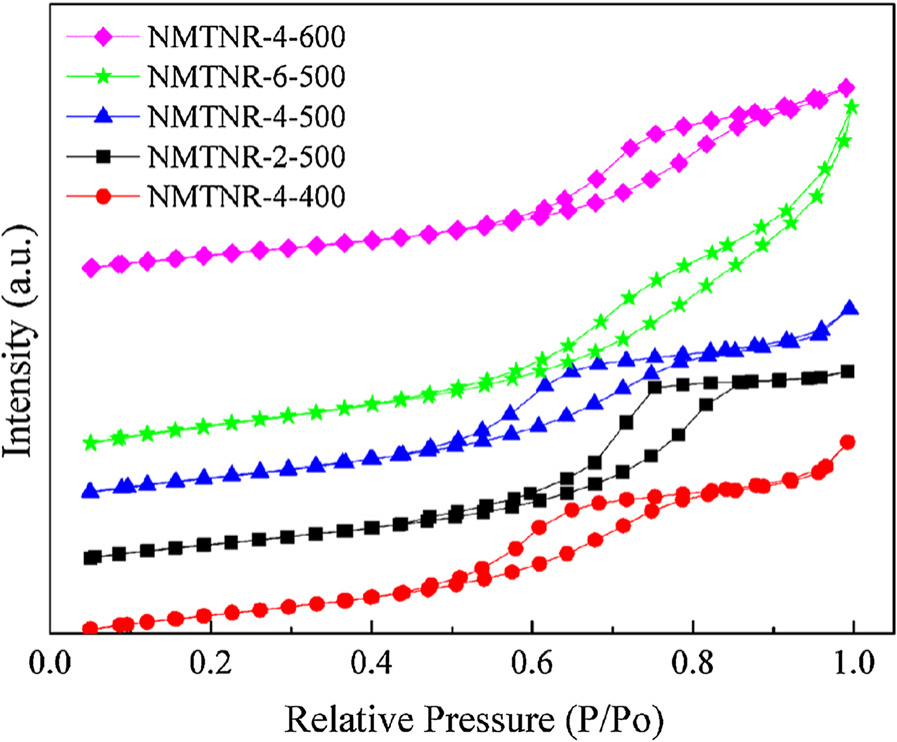
**N**_**2 **_**adsorption-desorption isotherms of N-doped mesoporous TiO**_**2 **_**nanorods.**

Figure 3 depicts the N_2_ adsorption-desorption isotherms of N-doped mesoporous TiO_2_ nanorods. The isotherms belong to the type IV with H2 hysteresis loop, indicating the existence of the porous structure [15]. According to the Brunauer-Emmett-Teller (BET) method, the specific surface areas for these samples (Table 1) are remarkably higher (76.1 to 106.4 m^2^ g^-1^) than that of Degussa P25 (50 m^2^ g^-1^). The Barrett-Joyner-Halenda (BJH) adsorption average pore diameters (4 V/A) and the pore volumes of the samples were also given in Table 1. It could be observed that with the increase of N proportion, the specific surface area and the pore volume was increased. The BJH adsorption average pore diameters were in the range of 5 to 10 nm.

**Figure 4:**
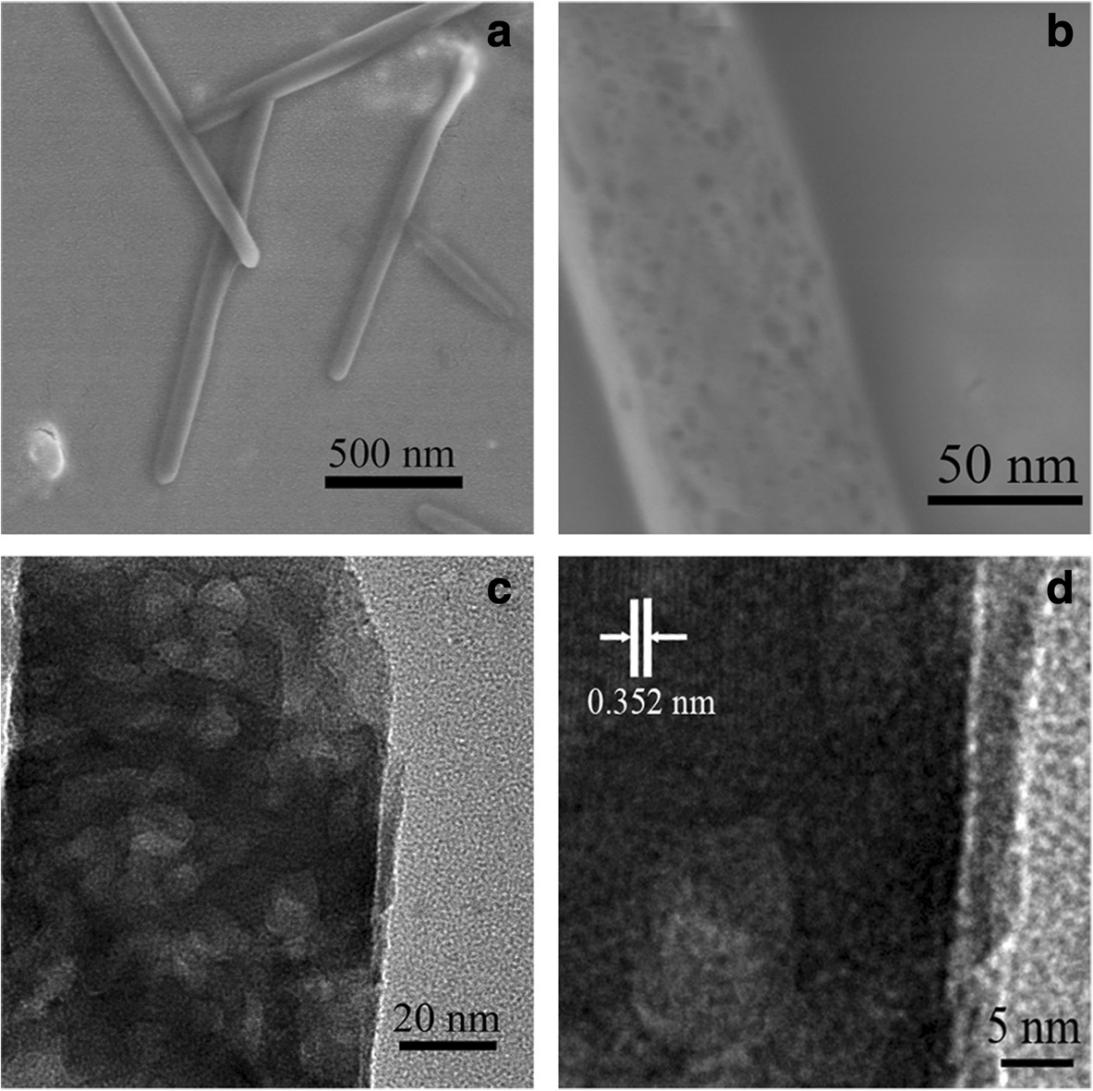
SEM (a, b), TEM (c), and HRTEM (d) images of NMTNR-4-500.

SEM, TEM, and HRTEM images of the sample NMTNR-4-500 are shown in Figure 4. It can be observed that the sample is made up of several nanorods with an average length of *ca*. 1.5 μm and a cross section diameter of *ca*. 80 nm. As shown in Figure 4b,c, the N-doped TiO_2_ nanorods are mesoporous structure. The corresponding HRTEM image is displayed in Figure 4d which proves the coexistence of mesoporous structure and a high crystallinity. The pore diameter is in the range of 5 to 10 nm, which is consistent with the N_2_ adsorption-desorption results (Table 1). The spacing of two neighboring parallel fringes is around 0.35 nm, which matches well with the *d* spacing between adjacent (101) crystallographic planes of anatase phase [16].

**Figure 5:**
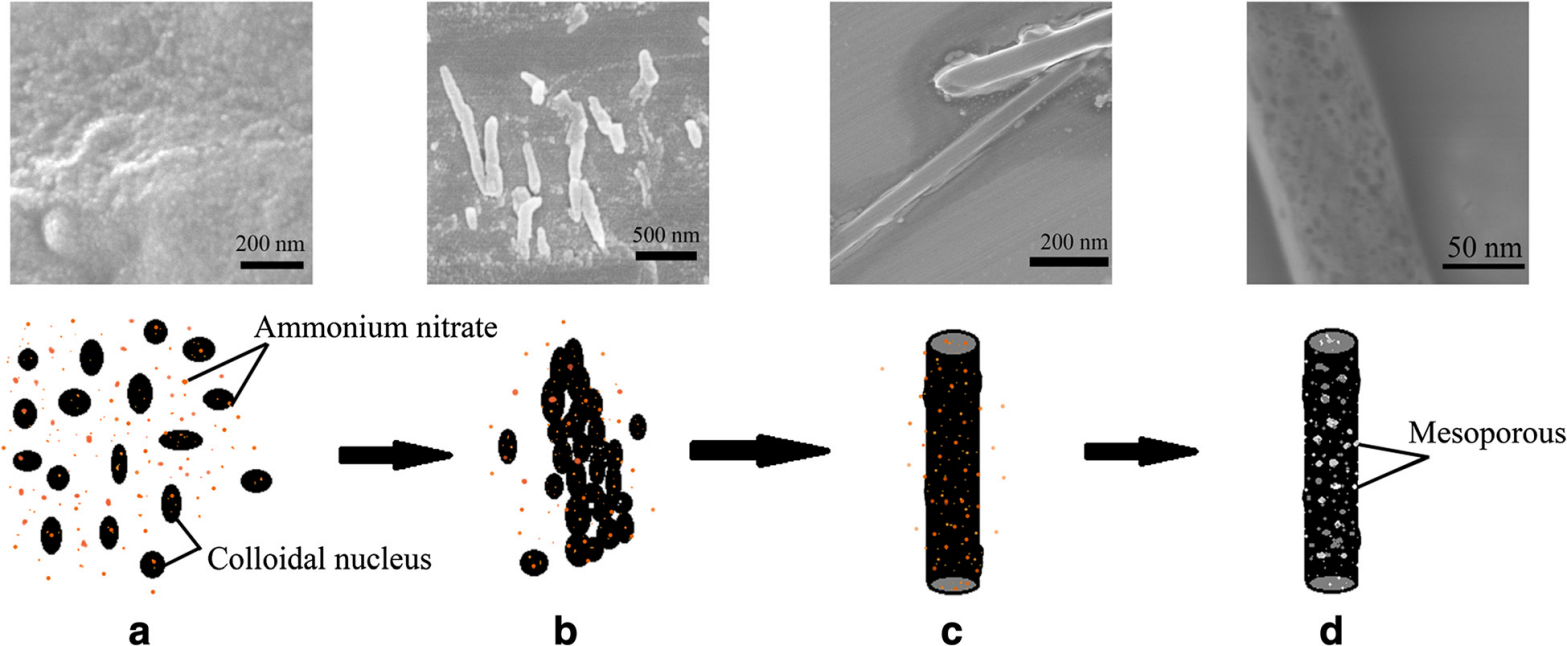
**The schematic illustration for N-doped mesoporous TiO**_**2 **_**nanorods. (a)** Formation of colloidal nucleus. **(b)** Rearrangement of colloidal nucleus. **(c)** Formation of rod-like structures. **(d)** Formation of N-doped mesoporous TiO_2_ nanorods.

Figure 5 shows a schematic illustration for the forming process of N-doped mesoporous TiO_2_ nanorods. This is based on the SEM observations of the N-doped mesoporous TiO_2_ nanorods at different periods and the existing mechanism of crystal growth [17]. In the experiment, vaporized molecules were transported with air into the reaction flask, resulting in the hydrolysis reaction of TBOT in the gas–liquid interface. Colloidal nucleus was formed in this process (Figure 5a). In addition, the rotation and the ball milling could improve the dispersion of colloidal nucleus in three-dimensional space. The colloidal nucleus rearranged to find a suitable place to reduce the surface energy (Figure 5b). Finally, TiO_2_ aggregates with rod-like structures were obtained (Figure 5c). When being annealed at 500°C, the ammonium nitrate attached on the surface of colloidal nucleus (see Additional file 1: Figure S1) was decomposed into N_2_, NO_2_, and H_2_O, which may result in the formation of mesoporous structure. At the same time, N_2_ and NO_2_ may provide the N source of as-prepared N-doped mesoporous TiO_2_ nanorods (Figure 5d).

**Figure 6:**
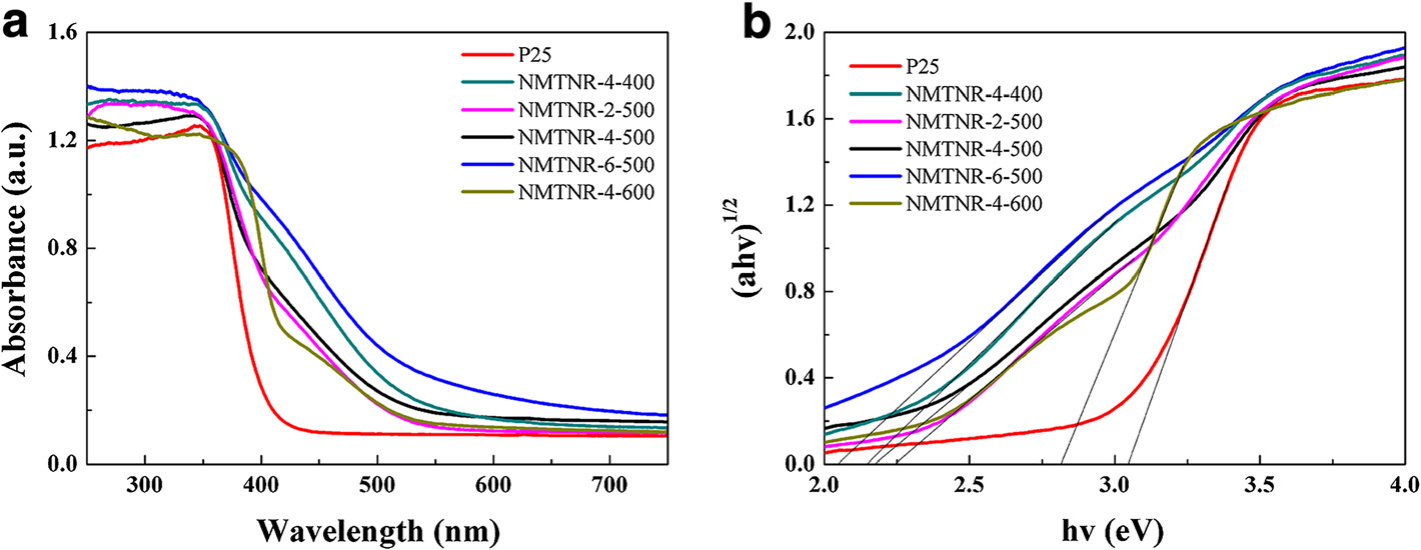
**UV–vis spectra and Kubelka-Munk function. (a)** UV–vis diffuse reflectance spectra for different samples and the respective Kubelka-Munk function for estimating the band gap energy (EBG) from variation of (*αhν*)^1/2^ with photon energy (*hν*) **(b)**.

The UV–vis absorbance spectra of as-prepared samples were shown in Figure 6a. It can be seen that the N-doped mesoporous TiO_2_ nanorods present a significant absorption in the visible region between 400 and 550 nm, which is the typical absorption feature of nitrogen-doped TiO_2_[18,19]. Kubelka-Munk function was used to estimate the band gap energy of the prepared samples. As TiO_2_ is an indirect transition semiconductor, plots of the (*αhν*)^1/2^ vs the energy of absorbed light afford the band gaps of the different samples (Figure 6b). The band gaps optically obtained in such a way were presented in Table 1. It reveals that the band gaps of N-doped mesoporous TiO_2_ nanorods are significantly narrower than that of P25, which is beneficial to the improvement of the photocatalytic efficiency.

**Figure 7:**
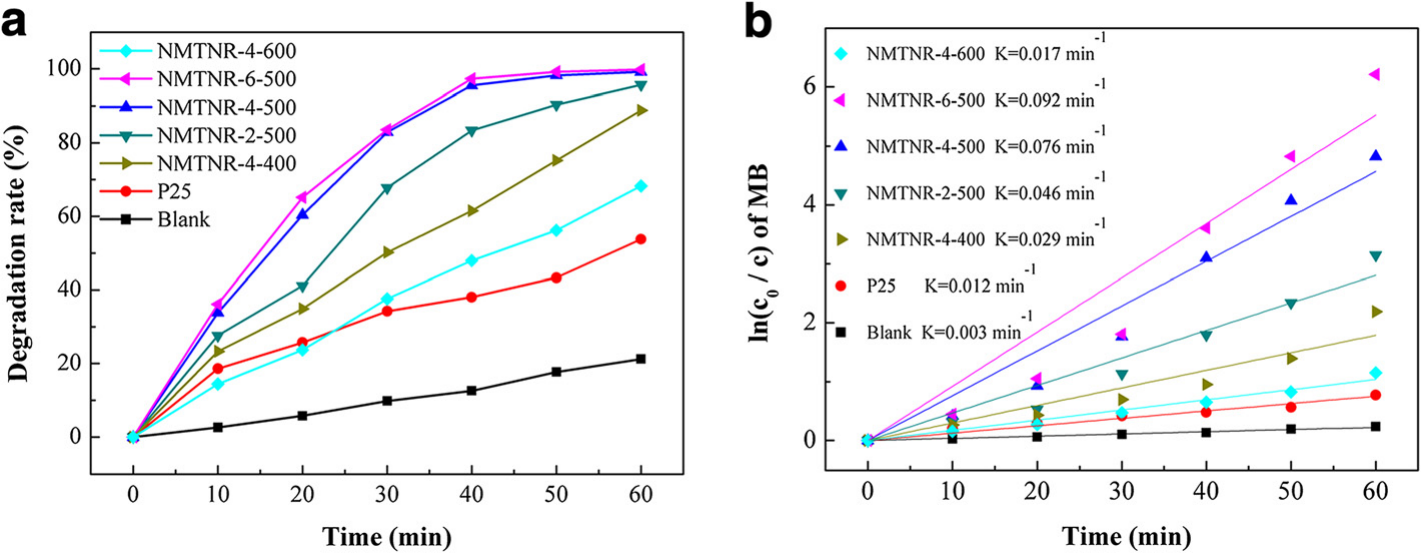
**Degradation curves of MB and plot of ln(*C***_**0**_**/*C*). (a)** The degradation curves of MB under visible light irradiation.** (b)** The plot of ln(*C*_0_/*C*) with irradiation time of visible light for different samples.

Figure 7a displays the degradation efficiency of MB versus irradiation time over different samples. A blank study (absence of catalyst) was carried out as a background check. For a comparison, P25 was investigated under the same conditions. It could be observed that without catalysts, only 21% of MB was degraded within 60 min. In contrast, the degradation efficiency of MB enhanced greatly in the presence of catalysts. The photocatalytic activity of the N-doped mesoporous TiO_2_ nanorods was much higher than that of the C-N co-doped rod-like TiO_2_ photocatalyst in our previous work [11]. The best catalytic efficiency was found in the sample NMTNR-6-500, which takes 60 min to degrade 99.8% MB in the solution, while the P25 degraded only 54% MB in the solution during the same time. Figure 7b shows a linear relationship between ln(*C*_0_/*C*) and the reaction time, indicating that the photodegradation of MB follows the first-order kinetics. The order of rate constants was summarized as follows: blank < P25 < NMTNR-4-600 < NMTNR-4-400 < NMTNR-2-500 < NMTNR-4-500 < NMTNR-6-500, which is consistent with the conclusions of photocatalytic degradation curves presented in Figure 7a.

Based on the data in Table 1, the excellent photocatalytic performance of N-doped mesoporous TiO_2_ nanorods might be explained by the following factors. Firstly, N doping could extend the spectral response to visible light and greatly improve the utilization of visible light [1,20]. Secondly, it is known that mesoporosity can improve surface adsorption capacity of the reactants due to the increased surface area [21,22]. It is obvious that with the increase of N proportion, the photocatalytic efficiency was improved. This may be resulting from the narrowed band gap and the enlarged surface area of N-doped mesoporous TiO_2_ nanorods. In addition, the calcination temperature also plays an important role in the catalytic efficiency. On the one hand, with the increase of the temperature, the grain size and band gap increased and the specific surface area decreased, which are responsible for the depress of photocatalytic activity. On the other hand, under lower temperature, TiO_2_ had a lower crystallinity, which results in the lower photocatalytic activity.

**Figure 8:**
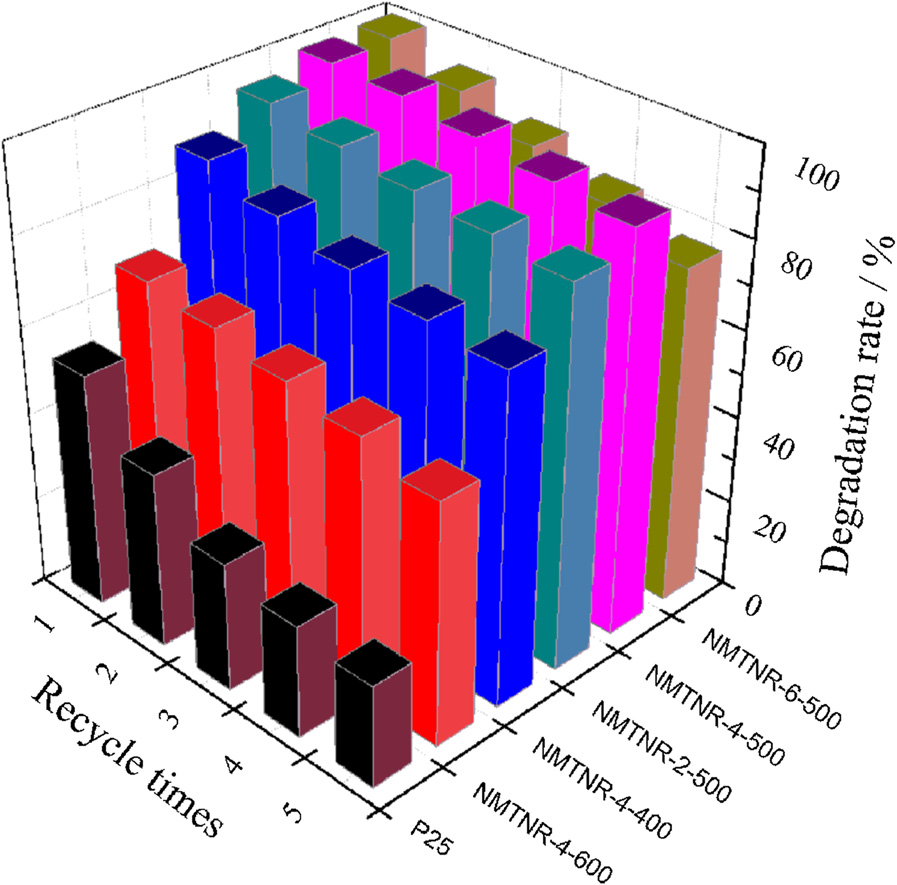
The photochemical stability of different samples.

To evaluate the stability of these photocatalysts, the repeated experiments for the degradation of MB were performed, and the results were shown in Figure 8. The reused N-doped mesoporous TiO_2_ nanorods maintained a higher catalytic activity than that of P25. Among all of the samples, NMTNR-4-500 showed the best photochemical stability, and it can still degrade 91.4% of MB within 60 min after five recycles. The rod-like structure takes many advantages, such as easy separation, recovery, and high recycle rate, which could enhance the stability of the photocatalyst [23,24]. However, it was noticed that the sample with the best catalytic efficiency (NMTNR-6-500) did not perform the best photochemical stability. This may be attributed to the destroyed nanorod structure caused by the excessive pores during the repeated use.

## Conclusions

In summary, the N-doped mesoporous TiO_2_ nanorods had been successfully fabricated by a template-free modified sol–gel approach. Ammonium nitrate was used to form the mesoporous structure and provided the source of N dopants. The average length and the cross section diameter of the as-prepared samples were *ca*. 1.5 μm and *ca*. 80 nm, respectively. The BJH adsorption average pore diameters were in the range of 5 to 10 nm. The mesoporous TiO_2_ nanorods doped with 6% theoretical molar ratio of N and annealed at 500°C showed the best photocatalytic performance. The photodegradation rate constant of this sample is 0.092 min^-1^, which is 7.6 times higher than that of P25. Furthermore, the rod-like photocatalyst can be easily separated and recycled, which could enhance the stability of the photocatalyst. The results provide useful insights for designing highly active photocatalyst.

## Competing interests

The authors declare that they have no competing interests.

## Authors’ contributions

The experiments and characterization presented in this work were carried out by XZ, ML, and GY. The experiments were designed by XZ, ZW, JL, and HJS. XZ, XL, and JJ analyzed and discussed the results obtained from the experiments. The manuscript was prepared by XZ. JL, HJS, and MZ helped with the draft editing. All authors read and approved the final manuscript.

## Supplementary Material

Additional file 1: Figure S1 IR spectra of TiO_2_ and NMTNR-4-500 before annealing. Click here for file
