# Supplementary material for: Simple fabrication of N-doped mesoporous TiO2 nanorods with the enhanced visible light photocatalytic activity
Source: Nanoscale Res Lett. 2014 Jan 16;9(1):34. doi: 10.1186/1556-276X-9-34 (PMC3901562; doi:10.1186/1556-276X-9-34)
Supplement: Additional file 1: Figure S1 — IR spectra of TiO2 and NMTNR-4-500 before annealing. [file 1556-276X-9-34-S1.doc]

**■**
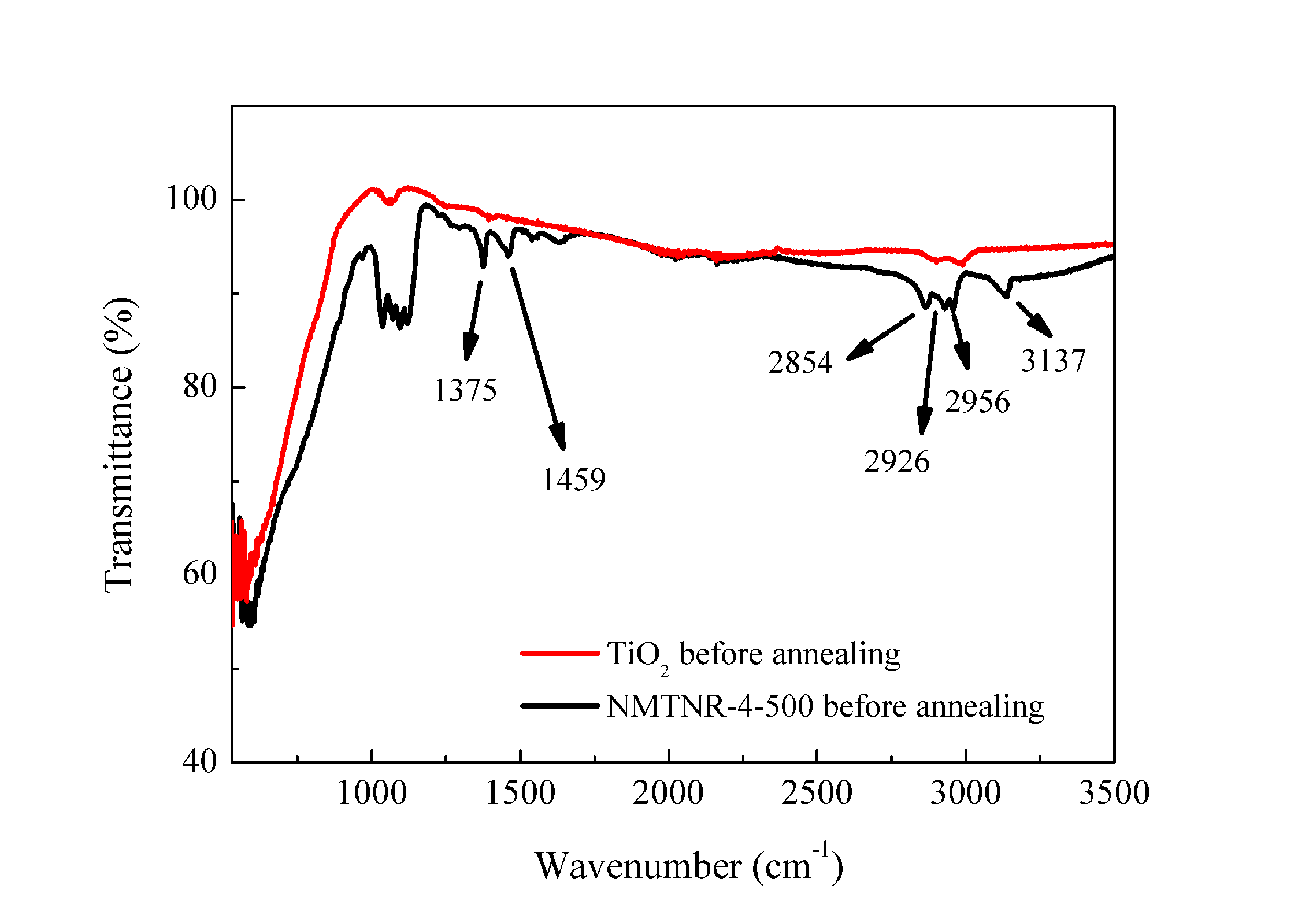


**Additional file 1:** Figure S1 IR spectra of TiO_2_ and NMTNR–4–500 before annealing.

Figure S1 depicts the IR spectra of TiO_2_ and NMTNR–4–500 before annealing. The attachment of ammonium nitrate on the surface of colloidal nucleus in NMTNR–4–500 before annealing could be evidenced by the absorbance peaks appeared at 1375 cm^–1^, 1459 cm^–1^, 2854 cm^–1^, 2926 cm^–1^, 2956 cm^–1^ and 3137 cm^–1^ [1, 2].

**References**

[1] Wu HB, Chan MN, Chan CK: **FTIR characterization of polymorphic transformation of ammonium nitrate.** Aerosol science and technology 2007, **41**(6): 581-588.

[2] Alavi S, Thompson DL: **Theoretical study of proton transfer in ammonium nitrate clusters.** The Journal of chemical physics 2002, **117**:2599-2608.
